# Differentiated processing of emotional cues in adolescents and young adults with ICD‐11 PTSD and complex PTSD after child abuse

**DOI:** 10.1002/brb3.2904

**Published:** 2023-02-07

**Authors:** Benjamin Iffland, Rebekka Eilers, Rita Rosner, Babette Renneberg, Regina Steil, Frank Neuner

**Affiliations:** ^1^ Department of Psychology Bielefeld University Bielefeld Germany; ^2^ Department of Psychology Catholic University Eichstätt‐Ingolstadt Eichstätt Germany; ^3^ Clinical Psychology and Psychotherapy Freie Universitaet Berlin Berlin Germany; ^4^ Department of Clinical Psychology and Psychotherapy, Institute of Psychology Goethe University Frankfurt Frankfurt Germany

**Keywords:** adolescents, complex posttraumatic stress disorder, EEG, electrocardiography, event‐related potential, heart rate, ICD‐11, posttraumatic stress disorder, psychophysiology

## Abstract

**Background:**

The recent update of the International Classification of Diseases 11th revision (ICD‐11) introduced the diagnosis of complex posttraumatic stress disorder (CPTSD) as a distinct entity from posttraumatic stress disorder (PTSD). Because psychophysiological alterations are a core diagnostic feature of PTSD and CPTSD, the aim of the current study was to examine potential distinctive patterns in cortical and cardiac responses to emotional words in adolescent and young adult patients with PTSD and CPTSD.

**Method:**

Event‐related potentials and heart rate responses were studied in 81 adolescent and young adult participants, of which 17 individuals were diagnosed with ICD‐11 PTSD and 32 individuals with CPTSD, each after childhood sexual and/or physical abuse. Thirty‐two individuals served as healthy controls. The paradigm consisted of a passive reading task with neutral, positive, physically threatening, and socially threatening words.

**Results:**

Differentiated early processing of emotional words was indicated by differences on P1 and left EPN components. Additionally, PTSD and CPTSD patients presented with specific patterns of heart rate responses to emotional words. In CPTSD patients, heart rate reactions to emotional words were more variable than in PTSD patients.

**Conclusions:**

These findings provide early evidence of differentiated cortical and cardiac response patterns in adolescent and young adult patients with CPTSD and PTSD, supporting a nosological distinction between PTSD and complex PTSD. However, due to small and unequal sample sizes, findings presented in the current study are preliminary and require future research.

## Graphical abstract

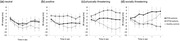

## 1 INTRODUCTION

Posttraumatic stress disorder (PTSD) has been associated with an accelerated processing of threat cues (Dalgleish et al., 2001). This response pattern has repeatedly been reported in analyses of event‐related potentials (ERPs) in response to trauma‐related pictures and descriptions as well as nonspecific negative pictures, negative emotional faces, and sounds (Javanbakht et al., 2011; Shvil et al., 2013). In‐line with this, distinct augmented late positive potential (LPP) amplitudes for responses to socially threatening words in adolescents and young adults diagnosed with PTSD after childhood sexual or physical abuse (CSA/CPA) have been indicated, whereas there were no differences in responses to socially threatening, physically threatening, positive, and neutral words in healthy controls (Klein et al., 2019). This pattern of emotional processing was accompanied by longer persisting cardiac responses to socially threatening words in young PTSD patients than in healthy controls (Iffland et al., 2020). Similarly, several studies reported larger cardiac responses to negative cues, for example, affective pictures and startling sounds, in adult patients with PTSD (Adenauer, Catani, et al., 2010; Buckley & Kaloupek, 2001; Pole, 2007).

However, less consistent with the threat signal hypothesis, adolescents and young adults with PTSD after CSA/CPA showed a blunted rather than increased cardiac response to physically threatening words (Iffland et al., 2020). In line, previous studies reported diminished reactions in specific subgroups of PTSD patients (Arditi‐Babchuk et al., 2009; Cuthbert et al., 2003; D'Andrea et al., 2013; Limberg et al., 2011; McTeague et al., 2010; Meyer et al., 2016). Particularly, attenuated physiological reactivity in PTSD patients was associated with dissociative symptoms (Lanius et al., 2002; Sack et al., 2012), multiple incident traumatization (Cuthbert et al., 2003; McTeague et al., 2010), and early developmental occurrence of traumatization (D'Andrea et al., 2013; Quevedo et al., 2010). Moreover, diminished reactivity has also been found in victims of childhood maltreatment (Heleniak et al., 2016; MacMillan et al., 2009). Correspondingly, differentiated heart rate reactions to emotional word categories were associated with different types of childhood traumatization (Iffland et al., 2020). Thus, the variability of reported physiological and attentional reactivities in PTSD patients may be associated with differences in onset, type, and chronicity of traumatic events, and severity and range of PTSD symptomatology in different subgroups of PTSD.

Clinical studies have consistently identified a dissociative, physiologically blunted subtype of PTSD (D'Andrea et al., 2013; Lanius et al., 2012; Van Der Kolk et al., 2005; Zucker et al., 2006), which had been recognized as part of the *DSM‐5* PTSD diagnosis (*Diagnostic and Statistical Manual of Mental Disorders*; American Psychiatric Association [APA], 2013). Similar to the revision of the diagnostic criteria of PTSD in DSM‐5 (APA, 2013), PTSD criteria have recently been reformulated and narrowed for the International Classification of Diseases 11th revision (ICD‐11) (World Health Organization [WHO], 2018). Although PTSD criteria consist of six core symptoms across the clusters re‐experiencing, avoidance, and hyperarousal, a new diagnosis of complex PTSD (CPTSD) was established requiring fully met diagnostic criteria of PTSD accompanied by disturbances of self‐organization that include affect dysregulation, negative self‐concept, and interpersonal problems (Cloitre et al., 2013; Maercker et al., 2013; WHO, 2018). Typically, CPTSD follows prolonged or multiple events, although a certain type or frequency of a traumatic event is not required for the diagnosis (Maercker et al., 2013).

With respect to children and adolescents, several studies have recently examined the consequences of changes to PTSD criteria (Danzi & La Greca, 2016; Sachser & Goldbeck, 2016; Sachser et al., 2017; Vasileva et al., 2018). Overall, ICD‐11 criteria seemed to be less sensitive than in other diagnostic manuals (Sachser & Goldbeck, 2016; Vasileva et al., 2018). However, latent class analyses of a broad range of trauma‐related symptoms provide two distinct patterns that are consistent with ICD‐11 PTSD and CPTSD (Perkonigg et al., 2016; Sachser et al., 2017). A recent comparison of prevalence rates of PTSD diagnoses according to DSM‐IV, DSM‐5, ICD‐10, and ICD‐11 in a treatment‐seeking sample of abused adolescents and young adults confirmed these results (Eilers et al., 2020). In total, 34% of the young patients who fully met DSM‐IV criteria did not meet ICD‐11 PTSD or CPTSD criteria. Mostly, this was due to missing features in the hyperarousal cluster despite reporting functional impairment. With respect to differences between ICD‐11 PTSD and CPTSD patients, Eilers et al. (2020) reported CPTSD to be more frequent in their sample. In addition, the CPTSD group showed higher scores for dissociation, depression symptom severity, and a number of comorbid diagnoses compared to participants with DSM‐IV PTSD, but not in comparison to ICD‐11 PTSD. Therefore, they concluded that diagnosing and differentiating ICD‐11 PTSD and CPTSD might prove difficult because the specific CPTSD characteristics might also represent childhood behavior that does not necessarily result from traumatic events.

Psychophysiological alterations are a core diagnostic feature of PTSD (Langeland & Olff, 2008), and with respect to its diagnostic criteria, physiological alterations are also substantial in CPTSD. However, studies on psychophysiological alterations in children and adolescents with CPTSD and differences in the psychophysiology between young patients with PTSD and CPTSD are still scarce. To date, neuroimaging studies in adults have suggested that CPTSD is associated with more severe neural correlates than PTSD (Marinova & Maercker, 2015). Structural brain abnormalities in CPTSD patients have been found to be more extensive than, and distinctive from, abnormalities in patients with PTSD after a single‐incident trauma (Thomaes et al., 2015). Predominantly, functional imaging studies revealed altered brain activation in CPTSD patients in brain regions involved in memory and emotion processing (Fonzo et al., 2016; Herzog et al., 2019; Thomaes et al., 2010, 2009, 2013). Moreover, CPTSD has been associated with alterations in the development of the neural networks involved in stress response regulation (Frewen & Lanius, 2006). With respect to attention, CPTSD was associated with a specific attentional bias toward trauma‐related words (Herzog et al., 2019). Particularly, findings indicated difficulties in dividing attention from negative words and impaired response inhibition for negative incoming information (Thomaes et al., 2013). However, the aforementioned research is limited as it did not examine patients fulfilling the CPTSD criteria proposed by the ICD‐11 (WHO, 2018) but rather patients with PTSD symptoms and complex PTSD symptoms consistent with the DSM‐IV‐TR Disorders of Extreme Stress Not Otherwise Specified (DESNOS) (APA, 2000) or DSM‐5 conceptualization (APA, 2013). Additionally, most studies compared CPTSD patients to control groups with or without a history of trauma exposure instead of the comparison of PTSD and CPTSD patients. Addressing this, a recent study provided the first evidence of distinctive neural processes during threat processing in CPTSD relative to PTSD patients (Bryant et al., 2020). Consistent with the assumption that CPTSD and PTSD differ with respect to disturbances in emotion regulation and self‐concept, PTSD and CPTSD patients showed differentiated insula and right amygdala activations. However, contrasting with previous evidence of reduced neural activations in dissociative PTSD (Lanius et al., 2010), CPTSD patients showed increased insula and right amygdala activations in response to emotional facial expressions when compared to PTSD patients. The authors related this contradiction to the stimuli used in their study and suggested that dissociative inhibition becomes most active in response to trauma‐related stimuli (Bryant et al., 2020). However, it remains unclear to what extent these early findings of a distinctive processing of threatening stimuli in PTSD and CPTSD can be transferred to other psychophysiological parameters.

At this point, it may be assumed that cortical and cardiac responses reflecting different stages of information processing differ in patients with PTSD and CPTSD. On the ERP level, potential candidates in the processing of emotional stimuli are the LPP, P100, and EPN (early posterior negativity) components. The LPP component is associated with motivated attention toward emotionally salient information (Hajcak et al., 2013). Previous studies found reduced LPP amplitudes for patients with PTSD during the processing of emotional facial expressions (DiGangi et al., 2017; MacNamara et al., 2013) indicating avoidance in the later information‐processing steps in PTSD patients. The P100 and EPN components were associated with early visual processing steps reflecting initial stages of attention orientation (Hofmann et al., 2009; Sass et al., 2010; Schupp et al., 2003). With respect to PTSD, prior studies reported diminished P100 amplitudes in patients with PTSD (Kounios et al., 1997), particularly in response to negative stimuli (Grégoire et al., 2018). Similarly, reduced EPN responses to threatening emotional pictures (Adenauer, Pinösch, et al., 2010) or faces (Felmingham et al., 2003) were reported in patients with PTSD when compared to healthy controls. Additionally, differential information processing in PTSD and CPTSD patients may be associated with differential cardiac responses to threatening stimuli. Functionally, initial decelerated cardiac responses were associated with orienting and heightened attention, whereas subsequent heart rate acceleration has been interpreted as reflecting preparation for action (Bradley et al., 2001).

Due to the limited number of studies examining physiological differences in patients with PTSD and CPTSD, particularly in children, adolescents, and young adults, the aim of the current study was to better understand potential differences in cortical and cardiac responses to differentially valenced emotional words in adolescent and young adult patients with PTSD and CPTSD. In doing so, ERP and Electrocardiography (ECG) data of ICD‐11 PTSD patients, CPTSD patients, and healthy controls collected within a larger treatment study protocol (Rosner et al., 2014, 2019) were reanalyzed. As stated above, original analyses were published recently (Iffland et al., 2020; Klein et al., 2019). In these studies, adolescents and young adults with a history of CSA and/or CPA who developed PTSD according to DSM‐IV‐TR (APA, 2000) were examined. For the purpose of the current study, diagnoses of all participants were rescored according to PTSD and CPTSD criteria proposed by ICD‐11 (WHO, 2018). CSA history has been highly associated with symptoms of CPTSD (Marinova & Maercker, 2015). Particularly, Hyland and colleagues (2017) found CSA to be the strongest risk factor for ICD‐11 CPTSD. Additionally, CSA also differentiated between ICD‐11 CPTSD and PTSD. Beyond CSA, the authors reported CPA to be an additional risk factor of CPTSD and cumulative exposure to childhood interpersonal trauma to be associated with CPTSD symptom severity (Hyland et al., 2017). Contrasting with recent studies using mainly images or faces as threat‐related or threat‐provoking stimuli to activate physiological reaction patterns, differentially valenced emotional words were used as stimuli in the aforementioned studies addressing the diverse history of traumatization in the sample of victims of CSA and/or CPA (Iffland et al., 2020; Klein et al., 2019). CPA and CSA represent interpersonal traumas, including the violation of the victim's personal and physical integrity that carry social stigma with them. Accordingly, it was suggested that physical threat cues (i.e., physically threatening words), as well as indicators of interpersonal or social threat (i.e., socially threatening words represented by swear words), were suitable to evoke trauma‐related physiological reactions that are associated with peri‐traumatic experiences (Iffland et al., 2020; Klein et al., 2019).

In particular, the goal of the present study was to investigate differential psychophysiological responses to physically threatening, socially threatening, positive, and neutral words in patients with PTSD and CPTSD. To reveal potential differences, different stages of information processing as reflected by the LPP, P100, and EPN components as well as heart rate responses were analyzed. More specifically, with respect to previous studies indicating diminished physiological reactions in specific subgroups of PTSD (e.g., D'Andrea et al., 2013; McTeague et al., 2010), we hypothesized that CPTSD was characterized by reduced psychophysiological responses to threatening words.

## 2 METHODS

### 2.1 Participants

The present data were collected within a larger treatment study protocol (for details, see Rosner et al., 2014, 2019). Participants of the patient groups were recruited from three German university outpatient clinics in Frankfurt, Berlin, and Ingolstadt. The healthy control group was recruited from a comprehensive school near Bielefeld, Germany. All participants were adolescents and young adults between the ages of 14 and 21 years and had sufficient knowledge of the German language.

The main criterion for inclusion for the patient groups was PTSD as a primary diagnosis following CSA and/or CPA after the age of three, according to the definition of the APA (2013) with an adapted diagnostic threshold of two instead of three avoidance symptoms. Patients were diagnosed using the German version of the Clinician Administered PTSD Scale for Children and Adolescents (CAPS‐CA; Steil & Füchsel, 2006) and the German version of the Structured Clinical Interview for DSM Disorders (SCID; Wittchen et al., 1997). Further, a stable psychopharmacological medication history was required for inclusion, meaning either no or continuous psychopharmacological medication during the previous 3 weeks. Living in stable conditions (no ongoing victimization, not homeless) and sufficient knowledge of the German language (clearly able to understand the information and instructions) were additional inclusion criteria. Exclusion criteria included acute suicidality within the previous 6 months, life‐threatening self‐harming behavior within the last 6 months, the presence of a substance‐related or organic mental disorder, pervasive developmental disorder, acute or lifetime diagnosis of a psychotic disorder, acute or lifetime diagnosis of a bipolar disorder, current diagnosis of substance dependence (abstinence less than 6 months) according to the Diagnostic and Statistical Manual of Mental Disorders, Fourth Edition (DSM‐IV‐TR; APA, 2000), mental retardation (IQ less or equal to 75), and simultaneous psychological or psychiatric treatment. For the healthy control group, exclusion criteria included any acute or lifetime diagnosis of an Axis I or Axis II mental disorder according to DSM‐IV‐TR. Clinical status of the healthy control group was assessed using a structured clinical interview that was conducted at the beginning of the experiment (SCID; Wittchen et al., 1997). The study was approved by the Ethics Committee of Bielefeld University, and the local independent review boards of Catholic University Eichstätt‐Ingolstadt, Goethe University Frankfurt, and Freie Universitaet Berlin.

To determine diagnoses of ICD‐11 PTSD and CPTSD, we adhered to the features recently published by the WHO (2018), supplemented with information from Maercker et al. (2013). ICD‐11 PTSD and CPTSD criteria had not yet been published, and standardized measures were not available at the time of data collection. Therefore, we mapped the features of ICD‐11 PTSD and CPTSD on matching items from CAPS‐CA, BSL‐23, and UPID with the diagnostic threshold for single items as described in the measures section (see Section 2.2). The six ICD‐11 PTSD features were based on CAPS‐CA items. For a good representation of the ICD‐11 reexperiencing feature, we mapped the symptom “intrusions or flashbacks” with two items (DSM‐IV symptoms B1 intrusions and B3 flashbacks). To operationalize “disturbances in self‐organization,” four features were based on CAPS‐CA. In addition, the symptom “diminished, defeated or worthless” was based on BSL‐23 (item “I feel worthless”) and the symptom “not feeling close to others” was based on UPID (item “feeling of detachment or estrangement from others”). A detailed description of the matched items was published in Eilers et al. (2020, online supplementary).

The final sample consisted of 81 participants (65 females, 80.2%), of which 17 individuals (21.0%) were diagnosed with ICD‐11 PTSD, 32 individuals (39.5%) with ICD‐11 CPTSD, and 32 individuals served as healthy controls (39.5%). The average age was 17.26 years (*SD* = 2.01). As reported in Table 1, 85.7% of the PTSD patients, 88.5% of the CPTSD patients, and 75.9% of the healthy controls indicated German as their native language. Nonnative speakers indicated that they had 1–17 years of experience with the German language (PTSD patients: 2–5 years; CPTSD patients: 1–12 years; healthy controls: 10–17 years).

**TABLE 1:** Participants’ characteristics and mean values on the assessments (*n* = 81)

	Adolescent and young adult PTSD patients (*n* = 17)	Adolescent and young adult CPTSD patients (*n* = 32)	Healthy controls (*n* = 32)	*p*
Age, *M* (*SD, range*)	17.65 (2.06, 14–21)	17.72 (2.53, 14–21)	16.59 (1.04, 15–20)	n.s.
Gender, *% female* (*n*)	94.1 (16)	84.4 (27)	68.8 (22)	n.s.^a^
Educational level (years), *M* (*SD*)	10.00 (1.84)	10.03 (1.31)	10.56 (.72)	n.s.
Maternal education level, *% without any certificate* (*n*)	15.4 (2)^b^	3.6 (1)^b^	3.4 (1)^b^	n.s.^a^
Maternal vocational education, *% without any* (*n*)	31.3 (5)^b^	14.3 (4)^b^	31.0 (9)^b^	n.s.^a^
Maternal unemployment, *% unemployed* (*n*)	31.3 (5)^b^ ^,1^	10.3 (3)^b^ ^,1^	3.2 (1)^b^ ^,2^	.006^a^
Paternal education level, *% without any certificate* (*n*)	0.0 (0)^b^	0.0 (0)^b^	0.0 (0)^b^	n.s.^a^
Paternal vocational education, *% without any* (*n*)	5.9 (1)^b^	0.0 (0)^b^	17.2 (5)^b^	n.s.^a^
Paternal unemployment, *% unemployed* (*n*)	18.2 (2)^b^ ^,1^	3.8 (1)^b^ ^,1,2^	6.7 (2)^b^ ^,2^	.021^a^
Citizenship, *% German* (*n*)	94.1 (16)	90.6 (29)	100.0 (32)	n.s.^a^
Migration background, *%* yes (*n*)	29.4 (5)	28.1 (9)	40.6 (13)	n.s.^a^
Native language, *% German* (*n*)	85.7 (12)^b^	88.5 (23)^b^	75.9 (22)^b^	n.s.^a^
Current residential situation, *% living with family members* (*n*)	75.0 (9)^b^ ^,1^	69.2 (18)^b^ ^,1^	100.0 (31)^b^ ^,2^	.012^a^
Intelligence quotient (CFT‐20‐R), *M* (*SD, range*)	101.81 (18.55, 78–125)^b^	99.13 (16.29, 79–140)^b^		n.s.
Beck Depression Inventory, *M* (*SD*)	26.47 (11.86)^1^	32.68 (12.95)^1^	6.47 (6.10)^2^	<.001
Interviewer‐rated PTSD symptom load (CAPS‐CA), *M* (*SD*)	66.29 (21.33)	74.00 (19.47)		n.s.
Borderline symptomatology (BSL‐23), *M* (*SD*)	31.12 (19.89)	43.00 (19.38)		.049
Self‐rated PTSD symptom load (UCLA PTSD Reaction Index) *M* (*SD*)	39.94 (13.11)^1^	47.22 (8.91)^1^	14.00 (10.84)^b^ ^,2^	<.001
Intrusion, *M* (*SD*)	13.06 (4.26)^1^	14.22 (3.96)^1^	2.94 (4.08)^b^ ^,2^	<.001
Avoidance, *M* (*SD*)	13.88 (6.41)^1^	17.91 (5.77)^1^	5.33 (5.20)^b^ ^,2^	<.001
Arousal, *M* (*SD*)	13.00 (4.23)^1^	15.09 (2.67)^1^	5.72 (4.27)^b^ ^,2^	<.001
Trauma Symptom Checklist for Children				
Anxiety, *M* (*SD*)	12.24 (4.79)^1^	13.56 (4.91)^1^	3.74 (2.44)^b^ ^,2^	<.001
Depression, *M* (*SD*)	11.35 (5.72)^1^	14.56 (4.84)^1^	4.06 (3.40)^b^ ^,2^	<.001
Posttraumatic stress, *M* (*SD*)	15.47 (4.16)^1^	18.16 (5.49)^1^	4.00 (3.26)^b^ ^,2^	<.001
Sexual concerns, *M* (*SD*)	4.35 (3.44)	5.75 (5.07)	3.20 (3.75)^b^	n.s.
Dissociation, *M* (*SD*)	9.71 (5.53)^1^	12.03 (5.76)^1^	4.84 (3.80)^b^ ^,2^	<.001
Anger, *M* (*SD*)	8.29 (5.05)^1^	8.34 (5.22)^1^	4.55 (3.84)^b^ ^,2^	.003
Childhood trauma questionnaire, *M* (*SD*)	64.94 (22.56)^b^ ^,1^	56.14 (19.29)^b^ ^,1^	32.81 (8.74)^2^	<.001
Emotional abuse, *M* (*SD*)	14.69 (5.90)^b^ ^,1^	14.55 (5.88)^b^ ^,1^	7.16 (2.33)^2^	<.001
Emotional neglect, *M* (*SD*)	16.06 (6.60)^b^ ^,1^	13.55 (6.16)^b^ ^,1^	8.25 (3.38)^2^	<.001
Physical abuse, *M* (*SD*)	10.88 (4.88)^b^ ^,1^	9.86 (5.57)^b^ ^,1^	5.66 (1.41)^2^	<.001
Physical neglect, *M* (*SD*)	12.13 (6.20)^b^ ^,1^	9.34 (4.53)^b^ ^,1^	6.19 (1.42)^2^	<.001
Sexual abuse, *M* (*SD*)	11.19 (6.67)^b^ ^,1^	9.39 (5.35)^b^ ^,1^	5.56 (2.83)^2^	<.001

Abbreviations: CPTSD, complex posttraumatic stress disorder; PTSD, complex posttraumatic stress disorder.

^a^ Chi‐squared test.

^b^ Smaller sample size due to missing values; means in the same row sharing the same superscript numeral do not differ significantly from one another at *p* ≤ .05 based on the Bonferroni corrected Student's *t*‐test or Chi‐squared test post hoc comparisons.

Out of the PTSD sample, three individuals indicated that they were currently receiving a psychopharmacological treatment. Of these, two individuals indicated the use of antidepressants, and one indicated the use of a psychopharmacological medication but was not able to specify which kind. No further use of prescriptive medication except oral contraceptives was present in the PTSD sample. In the CPTSD sample, five individuals indicated that they were currently receiving a psychopharmacological treatment. Here, three individuals indicated the use of antidepressants. Of these, one indicated additional use of anxiolytic and neuroleptic medication. Additionally, one individual indicated the use of anxiolytic, hypnotic, and anticonvulsant medication. Finally, one participant of the CPTSD sample indicated the use of a psychopharmacological medication but was not able to specify which kind. No further use of prescriptive medication except oral contraceptives was present in the CPTSD sample. Table 1 presents participants’ means on the assessments.

### 2.2 Instruments

The presence and severity of PTSD in patients according to either to the DSM‐IV or the ICD‐10 criteria were determined by the CAPS‐CA (Nader et al., 1994; German version: Steil & Füchsel, 2006). The CAPS‐CA was developed to assess the frequency and intensity of PTSD symptoms on a scale ranging from 0 (never) to 4 (daily or almost daily) and from 0 (none) to 4 (extreme), respectively. Symptom severity was determined by the sum of frequency and intensity ratings (range 0–136). As suggested by the German interview guidelines (Steil & Füchsel, 2006), a symptom is recorded as present if frequency and intensity are both rated with at least 1 (meaning mild intensity and one or two occurrences in the course of the previous month).

Comorbidity was assessed with the German version of the SCID (First et al., 1994, 1997; Wittchen et al., 1997). In the healthy control group, only the SCID was administered. All interviews were conducted by Master‐level clinical psychologists trained in the application of the CAPS‐CA and SCID.

The University of California, Los Angeles PTSD Reaction Index (UPID; Steinberg et al., 2004; German version: Ruf et al., 2010) provided a self‐rating of PTSD symptoms. The UPID measures PTSD symptom frequency during the previous month with 28 items and a total score ranging from 0 to 68. Items are rated on a four‐point scale. To estimate CPTSD symptoms, a score of 3 (“much”) or higher was seen as confirmation of the clinically relevant presence of a symptom as suggested by Steinberg et al. (2004).

The Borderline Symptom List 23 (BSL‐23; Bohus et al., 2009) was administered in order to assess for the presence of symptoms of borderline personality disorder. The BSL‐23 is a self‐rating questionnaire consisting of severity ratings of 23 borderline symptoms during the previous week and a total score ranging from 0 to 92. Items are rated on a five‐point scale from 0 (not at all) to 4 (very much). In‐line with the threshold used for the UCLA PTSD reaction index, a symptom rated with a score of 3 (“much”) or higher was considered to be relevant for CPTSD diagnosis.

Trauma‐related symptoms were assessed using the German version of the Trauma Symptom Checklist for Children (Briere, 1996; Matulis et al., 2015). The German version of the childhood trauma questionnaire (CTQ; Wingenfeld et al., 2010) was applied to measure different types of childhood maltreatment (sexual abuse, emotional neglect, emotional abuse, physical neglect, and physical abuse). The items are rated from 1 (never true) to 5 (very often true) with a possible range of subscale scores from 5 to 25.

Comorbid symptoms of depression were assessed using the German version of the Beck Depression Inventory (BDI‐II; Hautzinger et al., 2006), a self‐report measure consisting of 21 items. Higher scores indicate more severe depressive symptoms (range 0–92).

In order to assess participants’ intelligence quotient and to control for mental retardation in the patient sample, the Culture‐Fair Intelligence Test (CFT‐20‐R; Weiß, 2006) was used. On four subscales, the CFT‐20‐R assesses basic or fluid intelligence with a minimum of cultural and educational bias. When participants were not able to conduct the CFT‐20‐R properly (e.g., due to disorder‐related concentration problems), school certificates were used to ensure that patients met the cognitive requirements to be included in the study.

### 2.3 Stimulus set and task

Electrocortical and cardiac reactions to emotional words were recorded using a passive reading paradigm. In the paradigm, 100 German nouns from 4 different affective categories (neutral, positive, physically threatening, and socially threatening) were presented. Although socially threatening words conveyed pejorative words (e.g., *freak*), physically threatening words were represented by physical threat (e.g., *bombs*). Descriptions of different actions, places, or conditions connected with a positive valence were used as positive words (e.g., *holidays* or *paradise*). Neutral words depicted things or places (e.g., *reading room* or *lamp*). The stimulus set had previously been used and elicited differential processing as a function of affective valence (Wabnitz et al., 2012). In this study, neutral words were rated as less arousing and valent, whereas socially and physically threatening words did not differ with respect to arousal and valence. Words were equated for word length and frequency across valence categories, except for socially threatening words. The latter were less frequent based on the CELEX database for written German. Furthermore, all words were rated for perceived threat for physical and social integrities (Wabnitz et al., 2012). A detailed description of the stimuli set was published by Iffland et al. (2020) and Klein et al. (2019).

The experiment consisted of six blocks. Within each block, all 100 words were presented in a randomized order. Each stimulus was shown for 4000 ms and was replaced by a fixation cross that was present for 500 ms. The inter‐trial interval was 500 ms. In order to maintain attention to the stimuli, participants were asked to respond to a magenta dot that appeared in 15% of the trials for 67 ms by pressing the right arrow key on a standard keyboard. The stimuli were presented on a 15‐in. computer monitor, approximately 60 cm in front of the participant's eyes and were shown in white letters (Arial font, 36 point) on a black background. For analyses, trials of each affective category were collapsed across all blocks.

### 2.4 Procedure

Prior to the laboratory session, participants were informed about study conditions and asked to provide informed consent. When under the age of 18, participants were provided with written information about the study and received an informed consent document that had to be signed by their legal guardians. After participants provided their consent, structured clinical interviews were conducted to screen for the inclusion and exclusion criteria of the study. If participants matched the requirements, they were asked to complete a sociodemographic questionnaire as well as the instruments described above. At the beginning of the laboratory assessment, sensors for EEG and peripheral physiological measurements were applied. Next, participants’ electrocortical and cardiac reactions to emotional words were recorded using the passive reading paradigm. After completion of the task and sensor removal, participants were debriefed thoroughly.

### 2.5 EEG recording and data reduction

EEG was recorded from 32 BioSemi active electrodes (i.e., ActiveTwo, BioSemi system, Amsterdam, the Netherlands). Recorded sampling rate was 512 Hz. Electrodes were fitted into an elastic cap following the BioSemi position system. Two separate electrodes were used as ground electrodes, a common mode sense active electrode and a driven right leg (DRL) passive electrode, which form a feedback loop that enables measurement of the average potential close to the reference in the A/D box (www.biosemi.com/faq/cms&drl.htm).

Preprocessing and statistical analyses were conducted using BESA (www.besa.de) and EMEGS, a MATLAB toolbox designed to perform analyses of EEG data (Peyk et al., 2011). Offline data were rereferenced to the average reference and then filtered with a forward 0.10 Hz highpass (6 dB/oct) and a zero‐phase 30 Hz low‐pass (24 dB/oct) filter. Filtered data were segmented from 100 ms before stimulus onset until 1000 ms after stimulus presentation. The 100 ms before stimulus onset was used for baseline correction. Eye movements were corrected using the automatic eye artifact correction method implemented in BESA (Ille et al., 2002). Additionally, trials exceeding a threshold of 120 μV were rejected. On average, 2.96% of all trials were rejected. There were no differences in retained trials between word categories, *F* (3, 222) = .63, *p* = .598, partial *η* ^2^ = .008, nor, importantly, was there an interaction between word categories and group, *F* (3, 222) = 1.43, *p* = .203, partial *η* ^2^ = .037.

Time windows and sensor clusters were selected in reference to Klein et al. (2019) but adjusted for the current sample. Time windows were segmented from 130 to 160 ms for the P1 component, from 240 to 380 ms for the EPN component, and from 400 to 650 ms and 650 to 900 ms for the LPP. For the P1, a parieto‐occipital cluster of five electrodes (PO3, PO4, O1, Oz, and O2) was selected. For the EPN, an occipital sensor cluster over the left hemisphere consisting of three electrodes was examined (left: P7, PO3, and O1). For early and late portions of the LPP, we used an anterior (F3, Fz, F4, FC1, and FC2) and a posterior (P3, Pz, P4, PO3, and PO4) electrode cluster of five electrodes each, including position and time as a factor (for details, see Klein et al., 2019). Because visual inspections of the collapsed localizers indicated that amplitudes of P1 and left hemisphere EPN peaked earlier when looking at patients’ data only, explorative analyses in PTSD and CPTSD patients were conducted using the same sensor clusters, but time windows segmented from 115 to 145 ms for the P1 component and from 190 to 245 ms for the EPN component.

### 2.6 ECG recording and data reduction

Peripheral psychophysiological data were recorded with the ActiveTwo BioSemi system (BioSemi, Amsterdam, the Netherlands). As per BioSemi's design, the ground electrode was formed by the common mode sense (CMS) active electrode and the DRL passive electrode. All bioelectric signals were digitized on a laboratory microcomputer using ActiView software (BioSemi) and monitored online for data quality. Data were recorded with a sampling rate of 512 Hz. ECG was recorded from Ag/AgCl sensors placed at the initial point of the sternum and at the distal end of the left costal arch.

Offline data inspection and manual artifact rejection for ECG were done in ANSLAB 2.6, a customized software suite for psychophysiological recordings (Blechert et al., 2016). R‐waves in the ECG data were identified automatically. Additionally, data were visually inspected for artifacts and edited accordingly, resulting in manual replacements of artifactual data points, editing of non‐recognized R‐waves, and exclusion of sections with high proportions of artifacts. The ECG signal was converted to the instantaneous interbeat interval (IBI) indicating the time in milliseconds (ms) between successive R‐waves of the electrocardiogram. In accordance with analyses reported by Iffland et al. (2020), cardiac responses were defined as averages across the 4000 ms word presentation relative to a 500 ms baseline before the onset of the words. Hereafter, positive values represent increasing IBIs (deceleration of cardiac responses), whereas negative values represent a decrease of the IBI (acceleration of the cardiac responses). These response patterns were analyzed in 500 ms segments. In the statistical analyses, two time segments were averaged in each case, so that the analyses included four segments of 1000 ms each.

### 2.7 Statistical analyses

All statistical analyses were carried out using the Statistical Package for the Social Sciences SPSS 25. Initially, EEG and ECG data of 75 patients were recorded. Out of these, 17 patients were diagnosed with ICD‐11 PTSD and 32 patients were diagnosed with ICD‐11 CPTSD. Those patients (*n* = 26) meeting neither ICD‐11 PTSD nor ICD‐11 CPTSD criteria were excluded from analyses. Additionally, the EEG and ECG data of 43 healthy controls were recorded. Out of these, data of 32 healthy controls were matched with the patient groups with respect to age, gender, and educational level. As a result, the present study sample consisted of 81 participants.

EEG scalp data eligible for analyses were present for 77 individuals (*n* = 16 PTSD patients, *n* = 29 CPTSD patients, and *n* = 32 healthy controls). EEG data were statistically analyzed with EMEGS. Several 3 (group: PTSD patients vs. CPTSD patients vs. healthy controls) × 4 (valence: neutral, positive, physically threatening, socially threatening) repeated measures analyses of variance (ANOVAs) were set up to investigate potential main and interaction effects of group and valence in time windows and electrode clusters of interest.

For analyses of the cardiac reactions to the emotional words, ECG data of 81 individuals (*n* = 17 PTSD patients, *n* = 32 CPTSD patients, *n* = 32 healthy controls) were eligible for analyses. Here, a 3 (group: PTSD patients vs. CPTSD patients vs. healthy controls) × 4 (valence: neutral, positive, physically threatening, socially threatening) × 4 (time: four 1000 ms segments spanning image presentation) ANOVA with repeated measures on time and valence was conducted using the instantaneous IBI as a dependent variable.

In both EEG and ECG data, additional post hoc ANOVAs were conducted separately for different valences and groups when necessary. Additionally, post hoc comparisons between groups were computed using the Tukey HSD test. Post hoc comparisons between valences were computed using *t*‐tests. Partial *η* ^2^ and Cohen's *d* were estimated to describe effect sizes (Cohen, 1988). According to Cohen (1988), partial *η* ^2^ values of 0.01 represent a small effect, 0.06 a medium effect, and 0.14 a large effect, respectively; Cohen's *d* values were considered large with a *d* of 0.80 or greater, moderate with a *d* of 0.50–0.79, and small with a *d* of 0.20–0.49. With respect to the restricted sample size in the present study, effects not reaching significance but indicating at least medium‐sized effects were further inspected. When Mauchly's test indicated the violation of the sphericity assumption, Greenhouse–Geisser corrections were applied and original degrees of freedom together with Greenhouse–Geisser *ε* are reported. All analyses were adjusted for multiple comparisons using false discovery rate (FDR) correction (Benjamini & Hochberg, 1995). A significance level of *p* ≤ .05 was used for ANOVAs and post hoc ANOVAs.

When ANOVAs and post hoc comparisons indicated differences between PTSD and CPTSD patients, analyses were also carried out as analyses of covariance (ANCOVAs) with the CAPS‐CA, the UPID index scores, as well as the CTQ sum score serving as covariates to control for the influence of PTSD severity, symptom load, and childhood trauma load. Similarly, all analyses were carried out as separate ANCOVAs with age, the CFT‐20‐R IQ‐score, and medication serving as covariates to control for the influence of age, intelligence, and medication. As the pattern of results did not change, covariates were dropped and ANOVAs are reported. However, additional significant effects of the covariates are reported when indicated.

## 3 RESULTS

### 3.1 EEG analyses

#### 3.1.1 LPP

**FIGURE 1:**
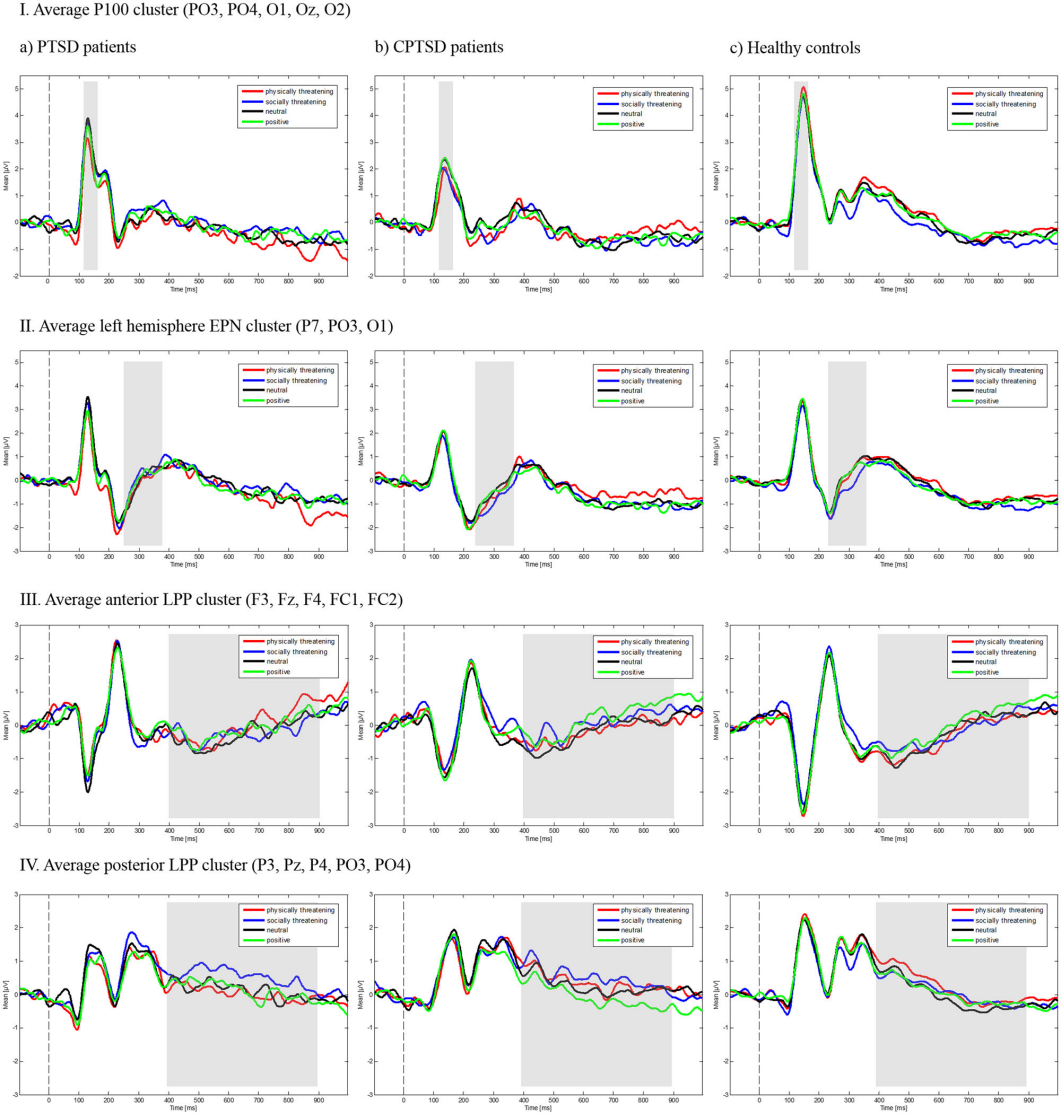
Average microvolt values for the event‐related potential (ERP) components for (a) posttraumatic stress disorder (PTSD) patients, (b) complex PTSD (CPTSD) patients, and (c) healthy controls.

In the early LPP time window, an initial repeated measures ANOVA showed a significant main effect of valence (*F* (3, 222) = 4.40, *p =* .005, partial *η* ^2^ = .056) (see Figure 1). Bonferroni adjusted post hoc *t*‐tests indicated that socially threatening words elicited significantly larger LPPs than neutral words (*t*(82) = 3.05, *p* = .003, Cohen's *d* = .33), whereas the other word categories did not differ (all *t*s < 1.95, all *p*s > .05/6, all Cohen's *d*s = .21). The main effect of region showed a significant effect (*F* (1, 74) = 10.40, *p* = .002, partial *η* ^2^ = .123). Further significant main or interaction effects were not found in the overall ANOVA (all *F*s < 1.16, all *p*s > .331, all partial *η* ^2^s < .030). Because of the significant effect of region, two additional 4 (valence) × 3 (group) repeated measures ANOVAs were conducted for the anterior and posterior electrode clusters separately. Here, neither the ANOVA in the anterior cluster (anterior: all *F*s < 1.83, all *p*s > .142, all partial *η* ^2^s < .024) nor in the posterior electrode cluster showed significant effects (posterior: all *F*s < 2.33, all *p*s > .075, all partial *η* ^2^s < .031). In the late LPP time window, the initial ANOVA with repeated measures did not show any significant effects (all *F*s < 2.06, all *p*s > .106, all partial *η* ^2^s < .028).

### 3.2 P1

A nonsignificant, but medium‐sized, main effect of group was found on the P1 component (*F* (2, 74) = 4.41, *p* = .016, partial *η* ^2^ = .106). Although PTSD and CPTSD patients did not differ (*p* = .959, Cohen's *d* = .09), CPTSD patients showed smaller P1 amplitudes than healthy controls (*p* = .018, Cohen's *d* = .71). *However, this difference did not remain significant after FDR correction*. Similarly, a medium‐sized, but nonsignificant, effect was found for the difference between PTSD patients and healthy controls (*p* = .107, Cohen's *d* = .64). The main effect of valence (*F* (3, 222) = .77, *p* = .509, partial *η* ^2^ = .010), as well as the interaction effect of valence and group (*F* (6, 222) = 1.74, *p* = .113, partial *η* ^2^ = .045), did not show significance.

Further explorative analyses in PTSD and CPTSD patients using a slightly earlier time window showed a small‐to‐medium sized, but nonsignificant, effect of group (*F* (1, 41) = 2.14, *p* = .151, partial *η* ^2^ = .050). PTSD patients showed larger P1 amplitudes than CPTSD patients. The main effect of valence (*F* (3, 123) = 1.99, *p* = .118, partial *η* ^2^ = .046) did not show significance, nor did the interaction effect of valence and group (*F* (3, 123) = .34, *p* = .800, partial *η* ^2^ = .008). Additionally, ANCOVAs with medication severity serving as covariate showed a medium‐sized effect of medication on the P1 (*F* (1, 40) = 2.43, *p* = .127, partial *η* ^2^ = .057), whereas the group effect still remained small‐to‐medium‐sized.

#### 3.2.1 EPN

The repeated measures ANOVA for the EPN component over the left hemisphere showed no significant effects (all *F*s < 1.33, all *p*s > .267, all partial *η* ^2^s < .018).

Further exploratory analyses of the EPN over the left hemisphere in PTSD and CPTSD patients applying an earlier time window revealed a medium‐sized, but nonsignificant, effect of group (*F* (1, 41) = 2.62, *p* = .113, partial *η* ^2^ = .060). CPTSD patients showed more pronounced EPN amplitudes than PTSD patients. There was no main effect of valence or interaction effect of valence and group (all *F*s < 1.33, all *p*s > .267, all partial *η* ^2^s < .031). Additionally, ANCOVAs with PTSD symptom severity serving as covariate showed medium‐to‐large effects of PTSD symptom severity on the left hemisphere EPN (UPID score: *F* (1, 40) = 5.96, *p* = .019, partial *η* ^2^ = .130; CAPS‐CA: *F* (1, 40) = 4.43, *p* = .042, partial *η* ^2^ = .100), whereas the group effect still remained small‐to‐medium‐sized.

### 3.3 ECG analyses

**FIGURE 2:**
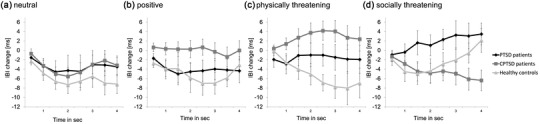
Means and standard errors of heart rate change in interbeat interval (IBI) (ms) across word presentation (4 s) in adolescent posttraumatic stress disorder (PTSD) patients, adolescent complex PTSD (CPTSD) patients, and healthy controls in response to (a) neutral, (b) positive, (c) physically threatening, and (d) socially threatening words. An increase in the IBI change scores indicates heart rate deceleration, and a decrease indicates heart rate acceleration.

The initial repeated measures ANOVA showed a significant interaction effect of valence × group (*F* (6, 234) = 3.12, *p* = .006, partial *η* ^2^ = .074). Because of the significant interaction effect, four one‐way post hoc ANOVAs were computed for each valence separately (neutral, positive, physically threatening, and socially threatening; see Figure 2).

**FIGURE 3:**
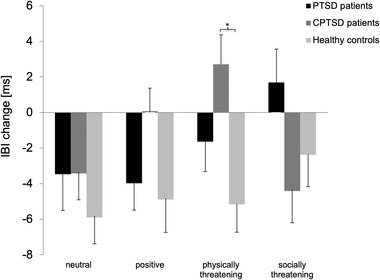
Means and standard errors of heart rate change in interbeat interval (IBI) (ms) relative to baseline for each group and for each word category (neutral, positive, physically threatening, and socially threatening). An increase in the IBI change scores indicates heart rate deceleration, and a decrease indicates heart rate acceleration. CPTSD, complex posttraumatic stress disorder; PTSD, complex posttraumatic stress disorder.

For physically threatening words, the one‐way ANOVA showed a significant main effect of group (*F* (2, 78) = 6.57, *p* = .002, partial *η* ^2^ = .144). Post hoc comparisons using the Tukey HSD test revealed that CPTSD patients’ heart rate reactions differed significantly from responses of healthy controls (*p* = .001, Cohen's *d* = .87), with CPTSD patients showing heart rate deceleration and healthy controls showing heart rate acceleration (see Figure 3). Although it did not reach significance, post hoc comparison of PTSD and CPTSD patients’ responses to physically threatening words showed a medium‐sized effect with CPTSD patients showing a more pronounced heart rate deceleration (*p* = .226, Cohen's *d* = .51). PTSD patients and healthy controls did not differ significantly (*p* = .371, Cohen's *d* = .43).

For socially threatening words, there was a medium‐sized, but nonsignificant, main effect of group (*F* (2, 78) = 2.48, *p* = .090, *η* ^2^ = .060). Although post hoc comparisons did not show significant differences between the two patient groups and healthy controls (healthy controls vs. PTSD: *p* = .303, Cohen's *d* = .44; healthy controls vs. CPTSD: *p* = .648, Cohen's *d* = .22), the post hoc comparison of PTSD and CPTSD patients indicated a nonsignificant difference in the reaction to socially threatening words of medium effect size (*p* = .073, Cohen's *d* = .73). PTSD patients showed decelerating heart rate reactions, whereas heart rates of CPTSD patients accelerated.

The repeated measures ANOVA for positive words showed a nonsignificant, but medium‐sized, effect of group (*F* (2, 78) = 2.95, *p* = .058, partial *η* ^2^ = .070). Here, PTSD patients and healthy controls showed a more pronounced acceleration of heart rates in reaction to positive words than CPTSD patients. Although these differences showed medium effect sizes, they were nonsignificant using the Tukey HSD test (PTSD vs. CPTSD: *p* = .258, Cohen's *d* = .58; healthy controls vs. CPTSD: *p* = .057, Cohen's *d* = .55). Heart rate reactions of PTSD patients and healthy controls did not differ (*p* = .931, Cohen's *d* = .10). Finally, the post hoc one‐way ANOVA did not show any significant effects in reaction to neutral words (all *F*s < 2.42, all *p*s > .093, all *η* ^2^s < .030).

Additionally, the initial repeated measures ANOVA showed a medium‐sized, but nonsignificant, main effect of group (*F* (2, 78) = 3.61, *p* = .032, partial *η* ^2^ = .085). Post hoc comparisons using the Tukey HSD test *indicated that mean heart rate reactions of healthy controls were significantly more accelerated than heart rate reactions of CPTSD patients (p* = .035, Cohen's *d* = .60). Overall heart rate reactions did not differ between PTSD and CPTSD patients (*p* = .924, Cohen's *d* = .15), and PTSD patients and healthy controls (*p* = .188, Cohen's *d* = .49). Additional main or interaction effects were not found in the initial repeated measures ANOVA (all *F*s < 2.03, all *p*s > .110, all partial *η* ^2^s < .040).

## 4 DISCUSSION

In a study of the neuronal and physiological responses to emotionally valenced words, we found evidence that adolescent and young adult participants with CPTSD can be differentiated from subjects with PTSD on ERP components reflecting very early stimulus processing (EPN and P1). In addition, PTSD and CPTSD patients presented with distinct patterns of heart rate responses to emotional words. Hence, the findings of the present study indicate that the neurophysiological alterations that are associated with PTSD and CPTSD (Bryant et al., 2020) can be identified in young patients. Given the fact that the patient groups did not differ with respect to the severity of PTSD symptoms and controlling for symptom severity did not weaken the group effects, differences of PTSD and CPTSD patients cannot be attributed to the PTSD symptom severity. However, the present analyses are restricted by small and unequal sample sizes and are based on estimates of effect sizes. Therefore, drawing definite conclusions from our preliminary findings is premature, and further research is needed.

### 4.1 ERP responses

With respect to ERP analyses, regardless of valence, PTSD as well as CPTSD patients showed smaller P1 amplitudes than healthy controls. This difference suggests a generalized, differentiated pre‐attentive processing in PTSD patients (Näätänen, 1990). A generalized diminished P1 activity in reaction to affective words had previously been reported by Kounios et al. (1997) and Grégoire et al. (2018). However, at an even earlier time window, PTSD patients showed larger P1 amplitudes than CPTSD patients. Previous studies have found that variability in the P1 component may depend on symptom severity (Zuj et al., 2017). Although the direction of effects needs further clarification, the present findings of distinctive P1 responses point to differentiated automatic attentional processing in PTSD and CPTSD patients. ERP responses between 100 and 200 ms have been proposed to reflect rapid emotional processing in terms of an early detection system of potential threats (Eimer & Holmes, 2002; Kounios et al., 1997; Zuj et al., 2017). Ford (2009) assumed that a dysregulation of this gain‐control mechanism is associated with disturbances of self‐regulation, particularly affect or emotion dysregulation, which is a core feature of CPTSD (WHO, 2018). Accordingly, impaired response inhibition was reported in CPTSD patients (Thomaes et al., 2013). However, contrasting with reports of a specific attentional bias toward trauma‐related words (Herzog et al., 2019), diminished P1 amplitudes were not valence‐specific in our study. Here, the use of standardized instead of trauma‐related stimuli may have weakened the valence effect on these early stages of emotional information processing.

Because P1 amplitudes are associated with the functioning of the amygdala and fusiform gyrus (Kounios et al., 1997; Rotshtein et al., 2010), our findings support previous reports that CPTSD and PTSD patients differ with respect to a threat‐processing network (Bryant et al., 2020; Thomaes et al., 2009) that includes, among other structures, the amygdala and the fusiform gyrus (Bryant et al., 2020; Thomaes et al., 2012, 2009). The greater activation of this network may result in the emotion dysregulation symptoms in CPTSD patients (Bryant et al., 2020). The present study further supports this assumption by indicating stronger EPN responses in CPTSD patients. Next to the P1, the EPN has been suggested to be linked to amygdala activity and to facilitate the processing of fear stimuli (Herrmann et al., 2007; Van Strien et al., 2014). However, although both P1 and EPN are associated with initial stages of attention allocation (Schupp et al., 2003; Zhang et al., 2014), divergent response patterns on the ERP components in the present study may reflect different stages in emotional word processing (Zhang et al., 2014). Although the P1 component has been thought to reflect a stage of differentiating nonthreatening and potentially threatening information, the EPN is thought to reflect an emotional/nonemotional discrimination stage of word processing (Zhang et al., 2014). Our findings indicate that different profiles of posttraumatic stress symptomatology modulate both early stages of emotional word processing.

There were no differences between PTSD and CPTSD patients with respect to LPP amplitudes in reaction to differently valenced words. This may indicate similar sustained attention and deliberate processing of emotional stimuli in both groups (Hajcak et al., 2012; Schupp et al., 2006; Weinberg & Hajcak, 2011) which has been associated with the symptom of avoidance (DiGangi et al., 2017; MacNamara et al., 2013). Accordingly, the patient groups did not differ with respect to self‐rated symptoms of avoidance in the present study. In‐line with Bryant et al. (2020) who argued similarly with respect to a lack of difference between CPTSD and PTSD in functional connectivity, it may be speculated that later components reflecting more evaluated attentional processes are increasingly associated with more nonspecific symptoms, including all types of trauma symptoms or possibly even psychopathology across disorders (Javanbakht et al., 2011).

### 4.2 Heart rate reactions

In addition to differences in ERP responses, the present study presents preliminary evidence of differentiated heart rate reactivity in adolescent and young adult ICD‐11 PTSD and CPTSD patients. However, cardiac reactions were, to some extent, in contrast to the findings of ERP data. In particular, although differences on early ERP components were found to be irrespective of word categories, patient groups showed varying heart rate response patterns to physically threatening, socially threatening, and positive words. PTSD and CPTSD patients did not only show quantitative but also qualitative differences depending on word valence.

Heart rate reactions of CPTSD differed from both the PTSD as well as the healthy group. In response to physically threatening words, CPTSD patients responded with a deceleration of heart rate, which may be indicative of a more pronounced downregulation of the autonomic nervous system (ANS) through parasympathetic responses. With respect to the rather blunted HR response of CPTSD patients to positive words and ERP findings, it may be argued that this downregulation in CPTSD refers to a variety of incoming emotional information. Alternatively, heart rate deceleration after physically threatening words may reflect an orienting response (Graham & Clifton et al., 1966) that is parasympathetically influenced and responsive to threat intensity (Bradley et al., 2001). Consequently, heart rate findings would indicate increased perceptual intake and attention in CPTSD patients in response to physically threatening words (Bradley, 2009). Accordingly, Elsesser et al. (2004) reported a heart rate deceleration to generally aversive pictures in PTSD patients. Interestingly, PTSD patients in the Elsesser et al. (2004) study showed an accelerative heart rate response to trauma‐related pictures. With respect to the findings of CPTSD responses in the present study, it may be assumed that physically threatening words may have been perceived as generally aversive stimuli, whereas the socially threatening words may have been more trauma‐relevant in our sample of victims of CPA and/or CSA. In light of this argumentation, accelerated cardiac responses in the CPTSD group in response to socially threatening words were in line with findings of elevated heart rates in reaction to trauma cues in adult and child PTSD patients (Adenauer, Catani, et al., 2010; Buckley & Kaloupek, 2001; Elsesser et al., 2004; Pole, 2007; Saltzman et al., 2005; Scheeringa et al., 2004). It may be, however, that the persisting accelerated cardiac responses to socially threatening words in CPTSD patients also represent unresponsive or tonic immobility associated with tachycardia (Schauer & Elbert, 2010). Accordingly, dissociation has been shown to be more frequent in CPTSD than in PTSD (Eilers et al., 2020).

Since the ANS has been suggested to be a transdiagnostic biomarker of emotion dysregulation, particularly self‐regulatory functions (Beauchaine, 2015), greater variability in cardiac reactions may also reflect greater disturbances in self‐organization and emotion regulation in CPTSD patients. Accordingly, dysregulation of the ANS is assumed to be more profound in CPTSD. Most notably, the parasympathetical nervous system—which was mainly addressed in our assumptions—is strongly interconnected with previously mentioned neural networks associated with CPTSD (Bryant et al., 2020; Thayer et al., 2009). However, mechanisms that underly the divergent physiological reactivity in adolescent and young adult PTSD and CPTSD patients after CSA/CPA require further examination.

Although responses to neutral and positive words did not differ in healthy controls and PTSD patients, as expected, reactions to threatening words were accelerated in healthy controls. PTSD patients, however, showed a rather blunted reaction to threat cues (in that they did not differ from zero reactivity). Keeping in mind that participants of the present study were adolescent and young adult PTSD patients after CSA/CPA, heart rate responses to threatening words in PTSD patients were in line with blunted physiological reactivity to threat cues reported for specific groups of PTSD patients, including survivors of childhood trauma (Cuthbert et al., 2003; D'Andrea et al., 2013; Limberg et al., 2011; McTeague et al., 2010).

### 4.3 Limitations

The present study has several limitations. Our findings are based on a selective sample of patients fulfilling DSM‐IV criteria for a diagnosis of PTSD. Therefore, the comparison of diagnostic groups was exploratory. In addition, the small and unequal number of patients in each diagnostic group is a strong threat to validity and limits statistical power. Particularly, larger and equivalent samples of healthy controls and CPTSD patients were used to examine potential differences with a much smaller PTSD sample. Accordingly, effect sizes rather than statistical significance were used to evaluate main and interaction effects. The more frequent occurrence of ICD‐11 CPTSD than PTSD may be due to our selective sample of patients with a history of CSA/CPA, as other studies reported that CPTSD was most commonly associated with interpersonal violence and childhood trauma (Knefel & Lueger‐Schuster, 2013; Sachser et al., 2017). Accordingly, Eilers et al. (2020), who analyzed a portion of the same sample of patients as the current study, found about 50% of the sample meeting ICD‐11 criteria for CPTSD. Moreover, with the use of self‐ratings and a clinical interview designed for DSM‐IV, we can only provide an estimate of ICD‐11 diagnoses. Hence, differences in ICD‐11 PTSD and CPTSD in adolescents and young adults should be assessed using tailored measurements and larger sample sizes in future studies. Next, the SCID (First et al., 1994, 1997; Wittchen et al., 1997) was used to assess comorbidity in the patient groups and diagnostical status in the healthy control group. Because the SCID is not validated for the use in adolescents, following studies should apply semi‐structured clinical interviews tailored for this sample. However, diagnostic criteria for mental disorders in DSM‐IV and DSM‐5 do not differ for adolescents and adults. Therefore, the validity of diagnoses in the present study can be assumed. Additionally, the stimulus words used in the present study were not rated with respect to arousal and valence by the present sample. Hence, results may be affected by different evaluations of valence and arousal of the stimuli in PTSD patients, CPTSD patients, and healthy controls. In addition, another limitation may arise from the study design. In order to reassure that the participants pay attention to the stimuli, the participants had to react as fast as possible to a magenta dot that randomly appeared in 15% of all trials. However, as the trigger appeared infrequently, the passive reading paradigm may have become more like an intermittent reaction task. In doing so, the participants’ attention may have shifted unintentionally toward the dot and away from the emotional words, which may have weakened the potential to reveal psychophysiological differences in emotional processing. Additional modulations of psychophysiological responses by demands of the task, however, should have occurred unconditionally across conditions and groups. Moreover, exploratory analyses did neither show differences between groups in the completion of the attention task nor significant influences (main or interaction effects) of the attention task on any of the outcome variables. Next, some of the participants indicated that they were receiving psychopharmacological treatment and analyses revealed an effect of medication on the P1 amplitudes. However, the sample was not large enough, and psychopharmacological treatment was too infrequent to further analyze the impact of medication on emotional processing in the patient groups. Future studies aiming for a recruitment of samples without medication or increased sample sizes would allow for subgroup analyses depending on medication status. Furthermore, due to the small sample size and limited power of analyses, analyses of the present study were restricted because averaged responses over the six blocks were used and changes of responses over the course of blocks were not inspected. However, valence and group effects in the processing of emotional words were rather weakened with this procedure. Therefore, the relevance of the presented results is not limited. Finally, findings of ERP and cardiac responses were, in part, contrasting, which challenges the reliability of the present result. It may be speculated that the paradigm used in the present study accounted for this. Standardized non‐trauma‐related words were used as stimuli. It is likely that differentiated physiological reactions in CPTSD and PTSD patients may be more pronounced and able to provide clear evidence of neuro‐ and peripheral physiological differences when using idiosyncratic stimuli consisting of words or images that were collected with respect to individual trauma histories. Despite these limitations, this study provides early evidence regarding differentiated cortical and cardiac response patterns in adolescent and young adult patients with ICD‐11 CPTSD and PTSD. Moreover, because both patient samples consisted of individuals with a history of CPA/CSA, the present study is among the first to show that distinctive physiology patterns in CPTSD and PTSD are associated with CPTSD symptomatology rather than with experiences of childhood maltreatment.

## 5 CONCLUSION

The current study is in‐line with previous research showing distinctive physiological reactivity in PTSD and CPTSD patients (Bryant et al., 2020). Extending previous reports of differences in functionality, impairment, and symptoms (Cloitre et al., 2013; Perkonigg et al., 2016), findings of differentiated cardiac and cortical responses support the nosological distinction between PTSD and CPTSD. However, due to the small and unequal sample sizes, analyses and findings presented in the current study are preliminary and require future research.

## CONFLICT OF INTEREST STATEMENT

The authors declare that they have no competing interests.

### PEER REVIEW

The peer review history for this article is available at https://publons.com/publon/10.1002/brb3.2904

## Data Availability

The datasets used and/or analyzed during the current study are available from the corresponding author on reasonable request.
